# Comments on: Follow-up of women after gynecological cancer treatment

**DOI:** 10.61622/rbgo/2025rbgo62

**Published:** 2025-07-15

**Authors:** Celal Akdemir

**Affiliations:** 1 Ministry of Health Izmir City Hospital Department of Gynecologic Oncology İzmir Türkiye Ministry of Health Izmir City Hospital, Department of Gynecologic Oncology, İzmir, Türkiye.

Dear Editor,

The FEBRASGO position statement entitled "Follow-up of women after gynecological cancer treatment", authored by Silva Filho et al.^(1)^ and published in the Revista Brasileira de Ginecologia e Obstetrícia, represents a valuable effort to standardize clinical practice in this important area.

However, we believe the document could be further strengthened by elaborating on specific patient subgroups and decision-making frameworks that frequently present challenges in routine clinical settings.

Firstly, the statement does not provide follow-up recommendations for patients undergoing fertility-sparing treatment for endometrial cancer. This population is at increased risk of residual disease and early recurrence following conservative therapy. Although the 2023 ESGO/ESHRE/ESGE guidelines offer detailed follow-up strategies tailored to this group, their absence from the current document represents a significant omission.^(2)^

Secondly, although the recommendations are presented in both textual and tabular form, the lack of a symptom-based algorithm or flowchart (e.g., symptomatic vs. asymptomatic patients) may lead to ambiguity in clinical decision-making. Incorporating a visual flowchart could significantly enhance the clarity and practical utility of this already comprehensive guideline.

Furthermore, the document's suggestion that telephone-based follow-up should only be considered under limited circumstances is not fully aligned with contemporary patient monitoring models. In populations facing geographic or socioeconomic barriers, structured telephone or digital surveillance should be more explicitly supported to enhance accessibility and long-term care continuity. While the guideline mentions that remote follow-up may be appropriate for patients who have been "adequately educated" about recurrence symptoms, it does not define the content, format, or standards of such education. Yet structured patient education is a prerequisite for the safe and effective implementation of remote follow-up, and leading telehealth best practice frameworks consistently emphasize its central role in such models.^(3)^

We sincerely commend the authors and editorial team for this important contribution and hope our suggestions may help inform future updates and further strengthen the impact of this guideline.
