# Supplementary material for: Comments on: Follow-up of women after gynecological cancer treatment
Source: Rev Bras Ginecol Obstet. 2025 Jul 15;47:e-rbgo62. doi: 10.61622/rbgo/2025rbgo62 (PMC12266856; doi:10.61622/rbgo/2025rbgo62)
Supplement: Related texts

# Author's reply to comment on: Follow-up of women after gynecological cancer treatment

**Authors:** Agnaldo Lopes da Silva, Mariana Seabra Leite Praça, Matheus Eduardo Soares Pinhati, Laura Guimarães Castro, Renato Moretti-Marques, Angélica Nogueira-Rodrigues, Eduardo Batista Cândido

**Affiliations:** 1 Universidade Federal de Minas Gerais Faculdade de Medicina Belo Horizonte MG Brazil Faculdade de Medicina, Universidade Federal de Minas Gerais, Belo Horizonte, MG, Brazil.; 2 Hospital Israelita Albert Einstein São Paulo SP Brazil Hospital Israelita Albert Einstein, São Paulo, SP, Brazil.

Dear Editor,

We sincerely appreciate the insightful comments from the correspondents regarding the FEBRASGO Position Statement, *"Follow-up of Women After Gynecological Cancer Treatment"*.^(1)^ Their thoughtful feedback provides a valuable opportunity to clarify specific points and further strengthen both the scientific foundation and clinical applicability of our recommendations.

## Follow-up After Fertility-Sparing Treatment

We acknowledge the correspondents’ observation regarding the need for tailored follow-up protocols for patients undergoing fertility-sparing treatment for endometrial cancer. Our original statement focused primarily on surveillance following standard treatment modalities (surgery ± adjuvant therapy). Although fertility-sparing management represents an evolving and critical component of care, the development of comprehensive guidelines for this specific subgroup was considered beyond the scope of this general follow-up statement.

The 2023 ESGO/ESHRE/ESGE guidelines provide detailed recommendations for surveillance in this context, including biopsies at 3 and 6 months following complete response, continued endometrial assessments every 3–6 months until conception or definitive surgery, and the optional use of pelvic exams and imaging.^(2)^ Recognizing the importance of this patient population, we intend to incorporate such guidance in future updates or in a dedicated statement focused specifically on fertility-sparing strategies.

## Visual, Symptom-Based Algorithm

While our current statement highlights symptoms as the main trigger for diagnostic investigation and includes structured tables adapted from NCCN and ESMO guidelines, we concur that a visual flowchart could enhance its practical value and support clinical decision-making in busy care environments. This enhancement will be carefully considered in future revisions to improve accessibility and usability.

## Remote and Telephone-Based Follow-up

We also acknowledge the growing role of telemedicine in the care of cancer survivors. Our guideline recommended telephone-based follow-up for low-risk patients who are adequately informed about symptoms of recurrence. Indeed, there is increasing evidence supporting the feasibility and safety of telehealth follow-up, particularly when implemented using standardized protocols and structured symptom checklists.^(3–5)^

However, the applicability of telemonitoring in Brazil warrants careful consideration. The assumption that remote follow-up expands access for socioeconomically vulnerable patients may not hold true in practice across all settings. For example, national data indicate that regions with the highest cervical cancer mortality rates also face the most severe structural barriers, such as low physician density, limited access to radiotherapy, and high levels of poverty and illiteracy.^(6)^ For many women in these contexts, in-person follow-up remains essential due to digital exclusion and low health literacy. Moreover, Brazilian data also show that patients with private health insurance demonstrate significantly higher adherence to digital health tools, such as electronic symptom-reporting apps, compared with users of the public health system.^(7)^

## References

[1] Silva Filho AL, Praça MS, Pinhati ME, Castro LG, Moretti-Marques R, Nogueira-Rodrigues A (2025). Follow-up of women after gynecological cancer treatment. Rev Bras Ginecol Obstet.

[2] Rodolakis A, Scambia G, Planchamp F, Acien M, Di Spiezio Sardo A, Farrugia M (2023). ESGO/ESHRE/ESGE Guidelines for the fertility-sparing treatment of patients with endometrial carcinoma. Hum Reprod Open.

[3] Hunn J, Tenney ME, Tergas AI, Bishop EA, Moore K, Watkin W (2015). Patterns and utility of routine surveillance in high grade endometrial cancer. Gynecol Oncol.

[4] Xiao K, Yeung JC, Bolger JC (2023). The safety and acceptability of using telehealth for follow-up of patients following cancer surgery: a systematic review. Eur J Surg Oncol.

[5] Leitch M, Arshad A, Cohen PA, Allanson ER (2025). Patient-initiated follow-up in low-risk endometrial cancer after surgery: a systematic review. Int J Gynecol Cancer.

[6] Silva AL, Romualdo GR, Pinhati ME, Neves GL, Oliveira JA, Moretti-Marques R (2025). Exploring cervical cancer mortality in Brazil: an ecological study on socioeconomic and healthcare factors. Int J Gynecol Cancer.

[7] Jorge TW, Filho C, Campagnaro M, Cartolano L, Singh J, Sena K (2021). Electronic patient reported outcome in Brazil: insurance provider impact on adherence. J Clin Oncol.
